# Thermal–Fluid–Solid Coupling Analysis of Enhanced Heat Transfer and Thermal Stress in Shark Skin Micro-Rib Tubes

**DOI:** 10.3390/biomimetics11100712

**Published:** 2026-10-06

**Authors:** Zhengkai Jiang, Jichen Sun, Hongguang Xiao, Muzhen Li

**Affiliations:** 1 College of Nuclear Science and Technology, Naval University of Engineering, Wuhan 430033, China; m25182703@nue.edu.cn (Z.J.); x22182701@nue.edu.cn (M.L.); 2 Institute of Chemical Defense, Academy of Military Sciences, Beijing 102200, China; sunjc97@163.com; 3 School of Nuclear Science and Technology, Xi’an Jiaotong University, Xi’an 710049, China

**Keywords:** shell-and-tube heat exchanger, shark-skin-inspired microstructure, enhanced heat transfer, thermal–fluid–solid coupling, thermal stress distribution

## Abstract

A multi-field coupled numerical model integrating fluid flow, convective heat transfer, and thermal stress is established for a shark-skin-inspired V-shaped micro-rib heat exchange tube. Using the shear stress transport (SST) k-ω model, its thermal–hydraulic performance is compared with a smooth tube, revealing the mechanism of vortex-induced thermal boundary layer disturbance. Based on an L32 orthogonal array and range analysis, the sensitivity of seven geometric parameters to pressure drop and heat transfer power is quantified. A one-way thermal–fluid–solid coupling technique maps the non-uniform temperature field onto the solid domain and solves thermal strain and stress via thermoelastic constitutive equations. Results indicate that the shark skin structure induces dense prismatic vortices, improving the heat transfer coefficient by 21.7% while incurring a 245.4% increase in pressure drop. Among the parameters, hrib/P exerts the most significant influence. Thermal stress concentration occurs at the rib–root junctions. Under specific nuclear power plant seawater heat exchanger conditions, the maximum thermal stress is about 42 MPa, far below the TA2 (Grade 2 titanium alloy) yield strength, ensuring the design remains within the linear elastic safety range without plastic yielding.

## 1. Introduction

Shell-and-tube heat exchangers, as key heat exchange equipment in fields such as marine nuclear power systems, liquefied natural gas, and the petrochemical industry, have performance that directly affects the safety and operational efficiency of the entire system. With the advancement of bionics, an increasing number of bionic structures—such as those mimicking pitcher plants, fish scales, animal and plant blood vessels, respiratory systems, and the fractal structures of leaf veins—have been applied in heat exchangers [1,2,3]. However, the normal operating conditions of heat exchange tubes often involve relatively complex multi-physics coupling effects; therefore, analyzing their flow and heat transfer characteristics and mechanical properties is equally important.

The unique V-shaped micro-riblet structure on the shark skin surface has attracted much attention due to its excellent performance in drag reduction and near-wall turbulence induction [2]. Many scholars have conducted research on the heat transfer and flow characteristics of heat exchange tubes with shark skin structures. Huang et al. [4] pointed out that by drawing on the non-smooth ribbed surface of shark skin, the near-wall fluid structures can be effectively controlled and the friction drag reduced. Li al. [5] showed that shark skin microstructures effectively thin the thermal boundary layer and enhance near-wall disturbance by inducing secondary flows and helical vortices. Sun et al. [6], using large eddy simulation, demonstrated that micro-ribs in a rhombic discontinuous arrangement can increase the near-wall vorticity peak by 22.3%, thereby achieving a maximum increase of 56.7% in the heat transfer coefficient. In terms of performance evaluation, the performance evaluation criterion(PEC) of a louvered fin optimized by an artificial neural network can reach 1.1348 [7]. Despite the engineering challenge that fouling deposition can weaken the enhancement effect [8], the shark skin structure significantly improves the thermohydraulic comprehensive performance while maintaining low friction loss.

In existing research, scholars usually adopt numerical simulation or experimental methods to study the heat transfer efficiency and flow characteristics of heat exchangers, or to investigate their mechanical properties separately. However, considering the combined effects of fluid scouring, temperature difference, and high-pressure conditions, both the thermohydraulic characteristics and the mechanical properties are very important; therefore, both should be fully considered. With the development of thermal–fluid–structure coupling technology, it has become possible to study the complex interactions among the fluid, heat transfer, and solid structure in heat exchangers by means of numerical simulation. In the failure analysis of a feedwater heater, Li et al. [9] established a three-dimensional simulation model by integrating the condensation process, conjugate heat transfer, and one-way fluid–structure coupling. The study used STAR-CCM+ to handle the fluid dynamics and combined it with the finite element method to determine the stress distribution in high-risk areas, such as the tube support plates, successfully predicting fatigue failure and the remaining life of the heater over a period of 23–29 years. For the cooling panels of an electric arc furnace, Gharib Mombeni et al. [10] used the k-ε turbulence model and conjugate boundary conditions, revealing that the severe temperature gradient caused by fluid stagnation at the pipe bends is the main cause of thermal fatigue cracks. In terms of optimization design, Zhao et al. [11] proposed a thermal–mechanical coupling optimization strategy based on the response surface method for the cooling channels of a gas-cooled microreactor. By parameterizing the geometry with NURBS curves, they achieved the coordinated optimization of peak temperature and thermal stress while reducing the computational cost. Jamesahar et al. [12] used the fluid–structure coupling method to study the effects of membrane stiffness and fluid parameters on the membrane shape and the convective heat transfer inside the cavity, finding that the influence of the membrane’s weight and buoyancy on its final shape cannot be ignored and that the change in membrane shape affects the heat transfer.

Regarding the analysis of thermal stress distribution and thermal fatigue damage in equipment, studies have shown that the temperature gradient within the equipment and the mismatch in thermal expansion coefficients between materials are the core factors generating thermal stress [13,14]. In high-temperature equipment, such as heat exchangers, welded or brazed regions often exhibit significant stress concentration due to geometric discontinuity, with the peak Mises stress typically occurring in the base material near the interface [15]. When equipment is subjected to frequent temperature fluctuations, severe thermal stress cycles can lead to thermal fatigue damage, which in turn induces crack initiation and propagation, and may even cause structural stress corrosion cracking [16]. Numerical simulation results indicate that increasing the preheating temperature can reduce the peak residual stress from 480 MPa to 453 MPa, thereby alleviating damage [17]. In addition, elastic designs, such as helical tube structures, can effectively release thermal stress and avoid failure risks under extreme temperature differences [18]. These findings highlight the importance of integrating thermal stress management into the design and operation of high-temperature equipment, as both temperature gradients and geometric discontinuities must be carefully controlled to ensure long-term structural integrity.

Thus, for complex enhanced heat exchange tubes, it is necessary to combine thermal–fluid–structure coupling calculation with finite element structural analysis: the former is used to describe the flow, heat transfer, and interactions between fluid loads and the solid structure, while the latter is used to further evaluate the stress and deformation caused by temperature gradients, pressure loads, and thermal expansion mismatch of materials. Based on this understanding, Sun et al. [6] proposed a shark-skin-inspired biomimetic heat exchange tube and applied it to single-phase heat transfer enhancement research in seawater heat exchangers of nuclear power plants. Taking the proposed biomimetic heat exchange tube as the research object, this study compared its flow and heat transfer characteristics with those of a smooth tube through numerical simulation and analyzed the influence of micro-rib geometric parameters on heat transfer performance and pressure drop. Meanwhile, by combining thermal–fluid–structure coupling calculation with finite element analysis, the thermal stress distribution on the shark skin micro-rib surface under typical operating conditions was further investigated. This study not only examined the heat transfer enhancement mechanism of the bionic micro-rib structure, but also considered its structural mechanical response during operation, providing a research basis for the comprehensive evaluation of the thermohydraulic performance and structural safety of bionic heat exchange tubes.

## 2. Materials and Methods

### 2.1. Physical Model

**Figure 1:**
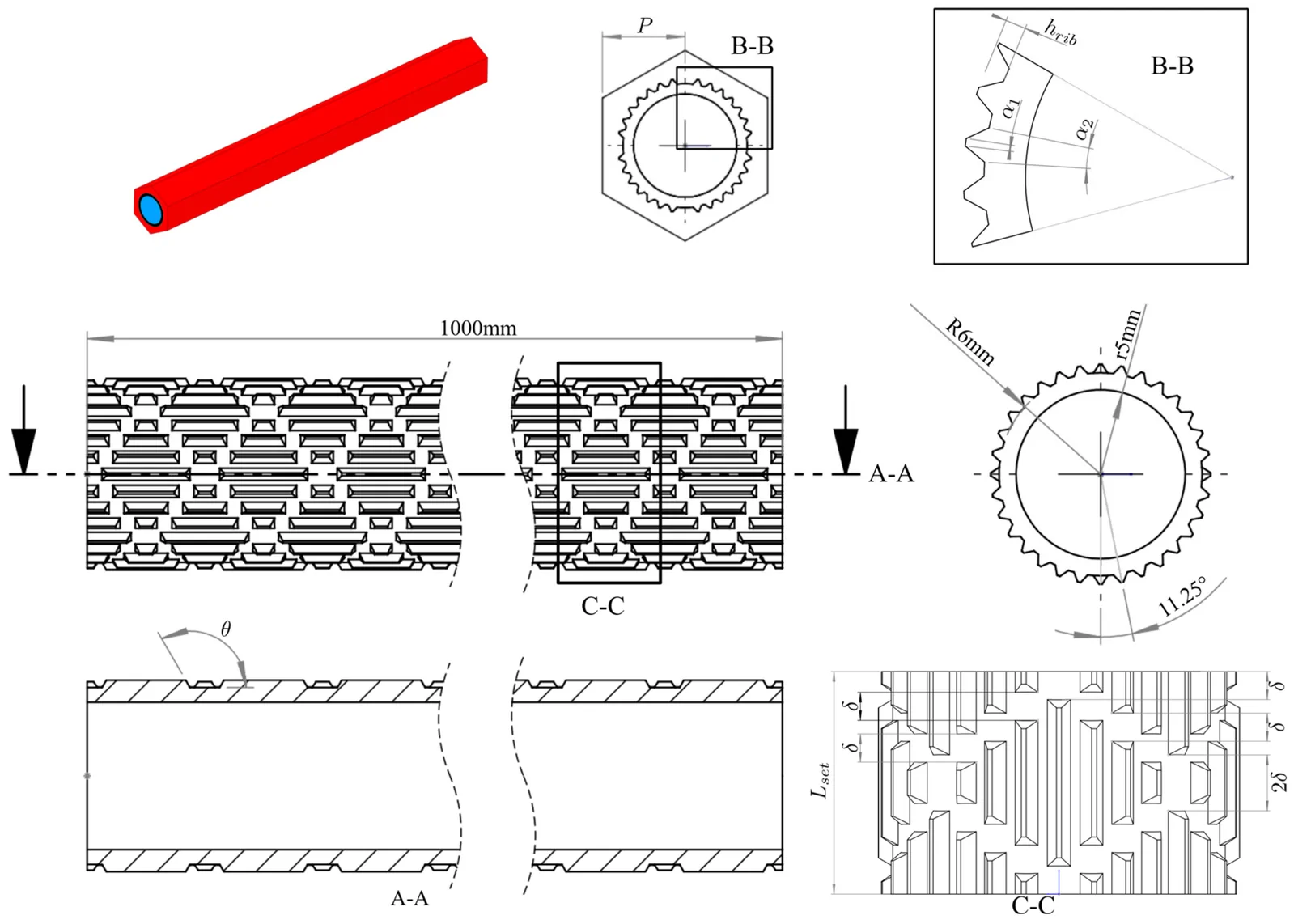
Design and characteristic parameters of the titanium alloy shark skin heat exchange tube.

Figure 1 is the design parameters of the titanium alloy shark skin heat exchange tube, with periodic micro-rib structures distributed on its surface in a staggered arrangement. The outer diameter of the heat exchange tube is Do = 12 mm, the inner diameter is Di = 10 mm, and the micro-ribs are uniformly distributed on the outer wall of the heat exchange tube. The key parameters characterizing the micro-rib geometry include rib height (*h_rib_*), rib group length (Lset), streamwise rib spacing (P), rib tip angle (α1), rib base angle (α2), and cross-sectional included angle (θ). The range of values for each parameter is determined according to actual manufacturing process constraints.

Physically, the problem considered in this study is steady conjugate heat transfer in a shell-and-tube seawater heat exchanger of a nuclear power plant. The representative hexagonal heat exchange unit consists of three coupled domains: the tube-side cold water domain, the TA2 titanium alloy tube wall (solid domain), and the shell-side hot water domain. Heat is transported from the shell-side hot fluid to the tube-side cold fluid through three mechanisms in series: convection from the hot fluid to the ribbed outer surface, conduction through the tube wall, and convection from the tube inner surface to the cold fluid, with the two streams in counterflow.

The following assumptions are adopted:

(i) The flow is steady, three-dimensional, incompressible, and single phase—boiling, cavitation, and any phase change are excluded.
(ii) The thermophysical properties of water are specified as follows: ρ = 998 kg·m^−3^, cp = 4183 J·kg^−1^·K^−1^, and k = 0.66 W·m^−1^·K^−1^, taken as uniform, and the dynamic viscosity is evaluated at each stream’s mean bulk temperature: μ = 9.81 × 10^−4^ Pa·s on the tube side and μ = 4.60 × 10^−4^ Pa·s on the shell side, giving molecular Prandtl numbers Pr = 6.22 and Pr = 2.92, respectively. The turbulent Prandtl number is set to Pr_t_ = 0.9 throughout.
(iii) Turbulence is modeled with the steady RANS SST k-ω closure—buoyancy, viscous dissipation, and thermal radiation are neglected.
(iv) All fluid–solid interfaces are no-slip, thermally coupled (conjugate) interfaces—no contact thermal resistance or fouling layer is considered.
(v) The hexagonal unit is treated as a representative element of the tube bundle. Symmetry is imposed on its lateral faces and translational periodicity in the axial direction, assuming the flow and thermal fields are fully developed.
(vi) The coupling is one way: the temperature and pressure fields computed in the fluid domain are mapped onto the solid domain, while the feedback of wall deformation on the flow is neglected. The TA2 alloy is modeled as a homogeneous, isotropic, linear-elastic material.
(vii) The convective heat transfer coefficient on the ribbed outer surface, h_o_, is defined with respect to the outer heat transfer area A_o_ as h_o_ = q″_w_/(T_w_ − T_ref_), where q″_w_ is the wall heat flux obtained from the conjugate fluid–solid interface, T_w is the local wall surface temperature, and T_ref_ is the bulk (reference) temperature of the shell-side fluid. The corresponding Nusselt number is Nu_o_ = h_o_ D_h,o_/k_f_, where D_h,o_ is the hydraulic diameter of the shell-side flow and k_f_ is the thermal conductivity of water evaluated at the film temperature.
(viii) The numerical simulations were performed using STAR-CCM+ 2402 (Siemens Digital Industries Software, Plano, TX, USA) for the conjugate heat transfer calculation in the fluid domains, and COMSOL Multiphysics 6.4 (COMSOL, Inc., Burlington, MA, USA) for the finite element analysis of the thermal stress in the solid domain. The two solvers were coupled in a one-way manner: the temperature and pressure fields calculated in the fluid domain were mapped onto the solid domain as the thermal load, while the feedback of wall deformation on the flow was neglected.

Accordingly, the boundary conditions and the surfaces on which they are imposed are velocity inlets and pressure outlets at the streamwise ends of the tube-side and shell-side domains, no-slip coupled walls on the tube inner surface and on the ribbed outer surface, an adiabatic condition on the outer boundary of the shell-side domain, symmetry on the lateral faces of the hexagonal unit, and translational periodicity on the two axial end faces. The quantitative values and a surface-by-surface specification are given in Section 3.1.

### 2.2. Governing Equations

#### 2.2.1. Fluid Dynamics Equations

In the field of fluid dynamics, the general governing equations are as follows:

(1) $$\nabla \cdot \bar{U}=0,$$

(2) $$\nabla \cdot \left(\bar{U}⊗\bar{U}\right)=-\nabla \cdot \left(\frac{\bar{P}I}{\rho }\right)+\nabla \cdot \left(\bar{\tau }+\tau_{\mathrm{RANS}}\right),$$

(3) $$\nabla \cdot \left(\bar{E}\;\bar{U}\right)=-\nabla \cdot \left(\frac{\bar{P}\bar{U}}{\rho }\right)+\nabla \cdot \left(\bar{\bar{\tau }}+\tau_{\mathrm{RANS}}\right)\;\bar{U},$$

(4) $$\tau_{\mathrm{RANS}}=2\mu_{\tau }S-\frac{2}{3}\left(\mu_{l}\nabla \bar{U}\right)I.$$

In the time-averaging process, unclosed terms are introduced. To close the system of governing equations, the SST k-ω turbulence model is introduced [19]:

(5) $$\nabla \cdot \left(k\bar{U}\right)=\nabla \cdot \left[\left(\nu +\sigma_{k}\frac{\mu_{l}}{\rho }\right)\nabla k\right]+\frac{P_{k}}{\rho }-\beta^{*}\omega k,$$

(6) $$\nabla \cdot \left(\omega \bar{U}\right)=\frac{\gamma P_{k}}{\mu_{l}}+\nabla \cdot \left[\left(\nu +\sigma_{\omega }\frac{\mu_{l}}{\rho }\right)\nabla \omega \right]-\beta \omega^{2}+2\left(1-F_{1}\right)\frac{\sigma_{\omega 2}}{\omega }\nabla k\cdot \nabla \omega ,$$

(7) $$\mu_{l}=\rho \frac{a_{1}k}{\max\left(a_{1}\omega ,\Omega F_{2}\right)},$$

(8) $$P_{k}=2\mu_{l}S:S-\frac{2}{3}\rho k\left(\nabla \cdot \bar{U}\right).$$

*P_k_* is the production of turbulent kinetic energy due to viscous forces, *k* represents the turbulent fluctuation energy per unit mass of fluid, and *ω* denotes the specific dissipation rate:

(9) $$\gamma =F_{1}\left(\frac{\beta_{1}}{\beta^{*}}-\frac{\sigma_{\omega 1}k^{2}}{\sqrt{\beta^{*}}}\right)+\left(1-F_{1}\right)\left(\frac{\beta_{2}}{\beta^{*}}-\frac{\sigma_{\omega 2}k^{2}}{\sqrt{\beta^{*}}}\right),$$

(10) $$F_{1}={\tanh\left(\min\left(\max\left(\frac{\sqrt{k}}{\beta^{*}\omega d},\frac{500\nu }{d^{2}\omega }\right),\frac{4\rho \sigma_{\omega 2}k}{CD_{\mathrm{k\omega}}d^{2}}\right)\right)}^{4},$$

(11) $$F_{2}={\tanh\left(\max\left(\frac{2\sqrt{k}}{\beta^{*}\omega d},\frac{500\nu }{d^{2}\omega }\right)\right)}^{2},$$

*σ_k_* = *F*_1_*σ_k_*_1_ + (1 − *F*_1_)*σ_k_*_2_. (12)

The above system of partial differential equations is discretized using a numerical solution framework based on the finite volume method. For the convection terms in each transport equation, a second-order central difference scheme is used for spatial discretization to ensure spatial accuracy in smooth flow regions. For the diffusion terms, an orthogonal decomposition method is adopted to handle their normal and cross components, thereby ensuring the physical consistency of the diffusion flux calculation, where the diffusion flux values at the interfaces are obtained via the least-squares gradient reconstruction method. To handle the coupling between the velocity and pressure fields, the SIMPLE algorithm is employed for iterative solutions.

The system of algebraic equations obtained after discretization is efficiently solved using the algebraic multigrid method, where the Gauss–Seidel iterative method is adopted as a smoother to eliminate high-frequency errors, supplemented by the preconditioned conjugate gradient method as an accelerator to improve convergence speed and numerical robustness under large-scale meshes.

#### 2.2.2. Solid-Mechanics Equations

After the fluid calculation converges, the temperature T and pressure P at each node of the shark skin external ribbed tube wall are extracted and mapped onto the solid domain grid nodes through a conservative interpolation scheme, i.e., adopting radial basis function interpolation for field variables such as temperature and displacement, and ensuring global flux conservation for heat flux and pressure.

The steady-state solid heat conduction equation is as follows:

(13) $$\nabla^{2}\bar{T}=0.$$

We describe the constitutive relationship between strain and stress of the external ribbed tube as:

(14) $$\varepsilon =\frac{1}{2}\left[\nabla u+{\left(\nabla u\right)}^{T}\right],$$

(15) $$\sigma_{\mathrm{ij}}=2\mu \;\varepsilon_{\mathrm{ij}}+\lambda \;\varepsilon_{\mathrm{kk}}\delta_{\mathrm{ij}}-\frac{\mathrm{E\alpha}}{1-2\nu }\Delta T\;\delta_{ij}.$$

Then, we post-process to obtain the von Mises equivalent stress:

(16) $$\frac{\partial \sigma_{\mathrm{ij}}}{\partial x_{j}}+F_{i}=0,$$

(17) $$\sigma_{\mathrm{vM}}=\sqrt{\frac{1}{2}\left[{\left(\sigma_{1}-\sigma_{2}\right)}^{2}+{\left(\sigma_{2}-\sigma_{3}\right)}^{2}+{\left(\sigma_{3}-\sigma_{1}\right)}^{2}\right]},$$

(18) $$\sigma_{\mathrm{vM}}=\sqrt{\frac{3}{2}S:S},$$

(19) $$S=\sigma -\frac{1}{3}\mathrm{tr}\left(\sigma \right)I,$$

**tr**(***σ***) = ***σ*_11_** + ***σ*_22_** + ***σ*_33_**. (20)

### 2.3. Numerical and Model Validation

**Figure 2:**
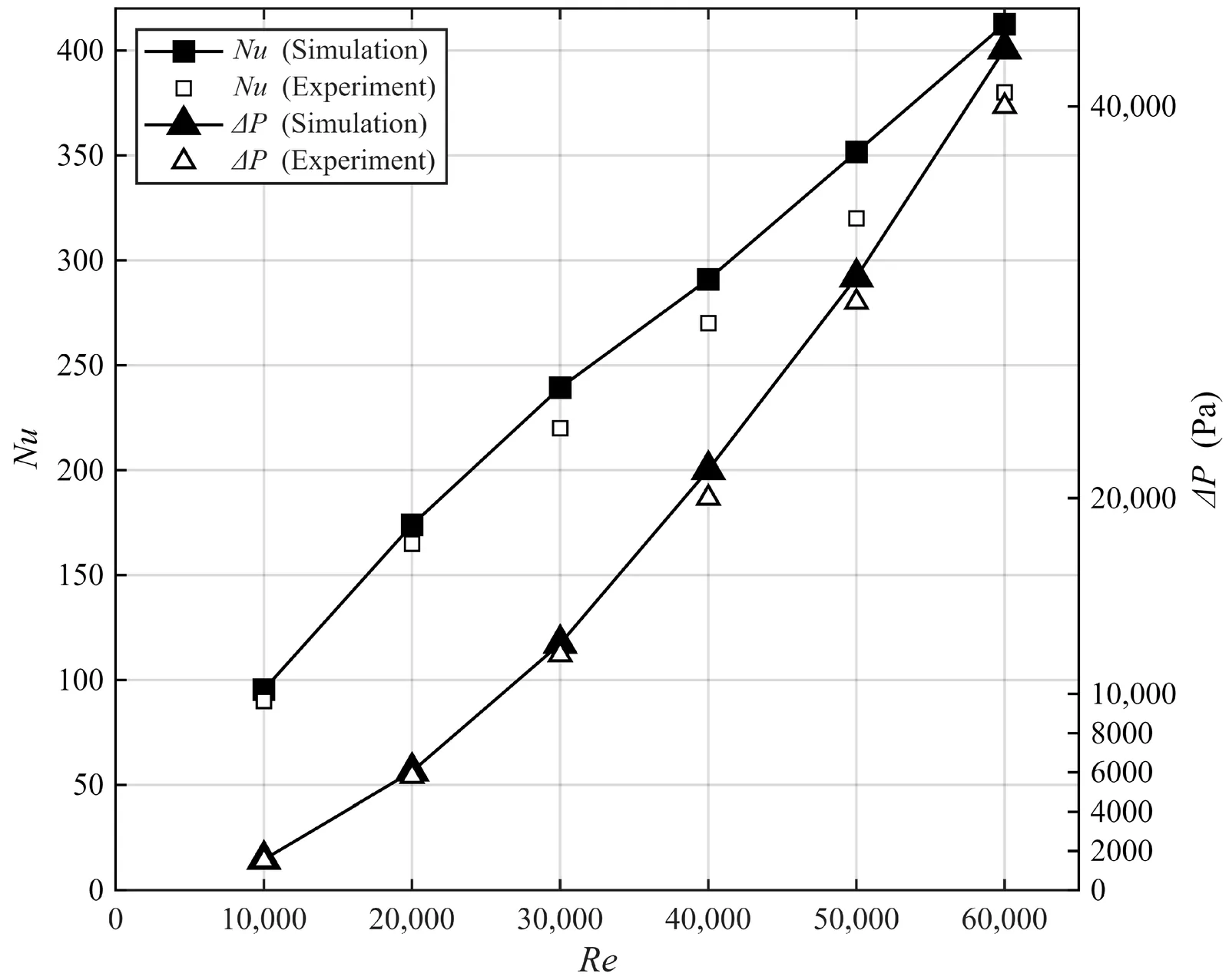
Comparison of the experimental data and the numerical simulation.

To validate the accuracy of the simulation results, the calculated results for the corresponding shark-skin-inspired heat exchange tube are compared with our previous experimental data [20]. As shown in Figure 2, the average deviation between the calculated and experimental Nusselt numbers is 6.34%, and that of the pressure drop per unit length is 5.92%. In that study, the flow rate, temperature, and pressure drop were measured with calibrated instruments, and the maximum relative uncertainties of the shell-side convective heat transfer coefficient and friction factor are 8.3% and 23%, decreasing to below 2.4% at the highest Reynolds number. The deviations, therefore, fall within the experimental uncertainty and may be partly attributed to the idealized boundary conditions and model assumptions adopted here. It can thus be considered that the numerical model is in good agreement with the experiment and is applicable to the present study.

To strike a balance between computational accuracy and efficiency, a grid independence verification was carried out on the shark-skin-inspired external ribbed tube model in this study. In view of the geometric characteristics of the biomimetic micro-groove structure, a meshing strategy combining unstructured polyhedral meshes with near-wall boundary layer meshes was adopted [21]. To accurately capture the near-wall flow field characteristics, 11 boundary layer mesh layers were set, and the first layer height was ensured to satisfy the requirement of *y*^+^ < 1 [22].

For the first type of external ribbed tube structure, four grid schemes with different densities were designed in this study, with total mesh counts of 2.14 million, 3.42 million, 4.86 million, and 6.15 million, respectively. The average Nu and ΔP were selected as the grid independence assessment indicators [23,24]. The validation results are shown in Table 1: when the mesh count increased from 4.86 million to 6.15 million, the relative change rate of the average Nu was only 0.62%, and the change rate of the pressure drop was below 0.45%.

According to the grid convergence index evaluation method, the discretization errors of all indicators have converged to a very small range [25]. After balancing computational accuracy and cost, the study finally decided to adopt the mesh scheme with approximately 4.86 million cells for all subsequent simulations of operating conditions.

## 3. Case Setup

### 3.1. Computational Domain and Boundary Conditions

To simplify the calculation, a representative hexagonal heat exchange unit in the tube bundle is selected as the computational domain, which includes three parts: the tube-side fluid domain, the tube wall solid domain, and the shell-side fluid domain, and periodic boundary conditions are used for modeling. The tube pitch P determines the overall size of the heat exchange unit. In this hexagonal unit, the shell-side and tube-side fluids are in counterflow, and the shell-side fluid temperature is higher than that of the tube side. Heat is transferred from the hot-side fluid to the cold-side fluid through the tube wall. This process includes convective heat transfer from the shell-side fluid to the tube wall, solid heat conduction within the tube wall itself, and convective heat transfer between the tube wall and the tube-side fluid.

**Figure 3:**
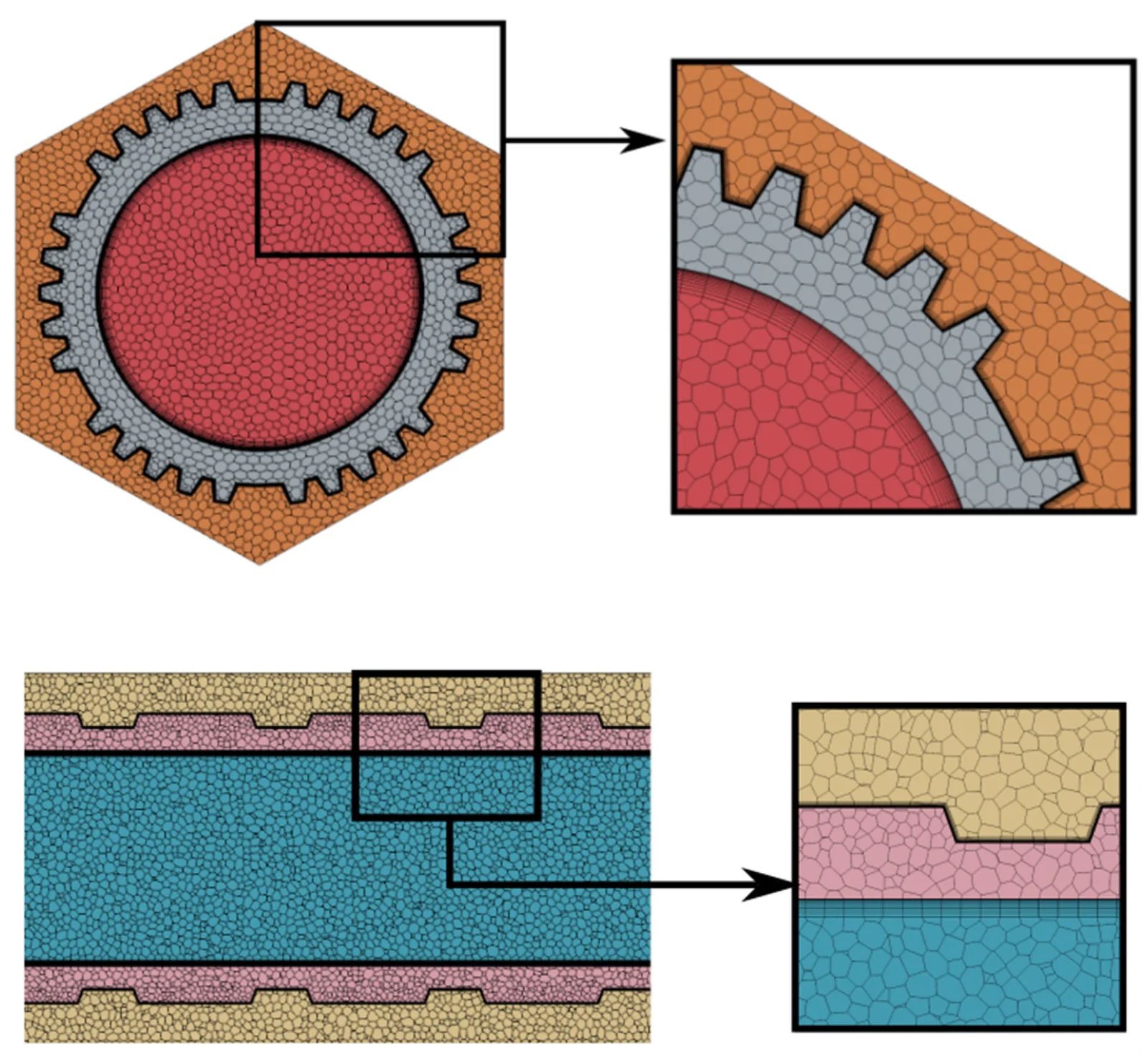
Computational domain and mesh of the shark skin heat transfer tube.

In this study, numerical simulations were conducted on the flow pressure drop and heat transfer rate of a shark-skin-microstructured heat exchange tube in a hexagonal channel. As shown in Figure 3, the entire computational domain includes the shell-side hot water section, the heat exchange tube wall, and the tube-side cold water section. The outer surface of the shell side was set as an adiabatic boundary, and symmetric boundary conditions were applied on the symmetry planes of the hexagonal heat exchange unit. The hot water on the shell side and the cold water on the tube side flow countercurrently. Periodic boundary conditions were applied at both ends in the main flow direction (axial), assuming that the flow and heat transfer have reached a fully developed stage in this direction. The heat exchange section was discretized using polyhedral meshes, and the interfaces between the tube side, tube wall, and shell side were configured with shared topological nodes to ensure mesh consistency on both sides of the interfaces. We set the tube-side inlet temperature to 20 °C and the velocity to 2.31 m/s, and set the shell-side inlet temperature to 60 °C and the velocity to 0.91 m/s.

In the calculation of thermal stress in the solid domain, the mesh of the tube wall was separately output. To ensure the convergence of the thermal deformation calculation for the ribbed structure, the tube wall was discretized separately using triangular meshes and refined. The axial temperature distribution in the solid domain was obtained through convective–conductive coupled heat transfer calculation, and the temperature field calculated from the fluid domain was mapped onto the corresponding nodes of the tube wall. Both end faces of the tube wall were set as adiabatic boundaries. When performing the thermal–structural coupled thermal stress analysis, normal displacement constraints were applied on the end faces to prevent rigid body motion. Because the pressure difference between the shell side and the tube side is small, the mechanical stress caused by the pressure is far below the material yield limit and negligible compared to the thermal stress. Therefore, to improve computational efficiency, this study adopts a one-way decoupling strategy, applying only the temperature field as the main load to the solid domain and temporarily disregarding the contribution of the pressure load to the total stress.

### 3.2. Design Variables of the Heat Transfer Tube

To extract the key geometric parameters affecting the thermohydraulic performance of the heat exchange unit, this study adopts a standard L32 orthogonal array to design the numerical experimental scheme. As shown in Table 2, a total of seven design variables are included in the analysis, and each variable is set to four levels within its value range. Through range analysis, the secondary factors with weak influence on the shell-side pressure drop and heat transfer rate can be identified. Based on the variable ranges listed in Table 2, the detailed L32 orthogonal experimental scheme and factor level allocation are shown in Table 3.

## 4. Results and Discussion

### 4.1. Heat Transfer Enhancement Mechanism of the Shark Skin Structure

**Figure 4:**
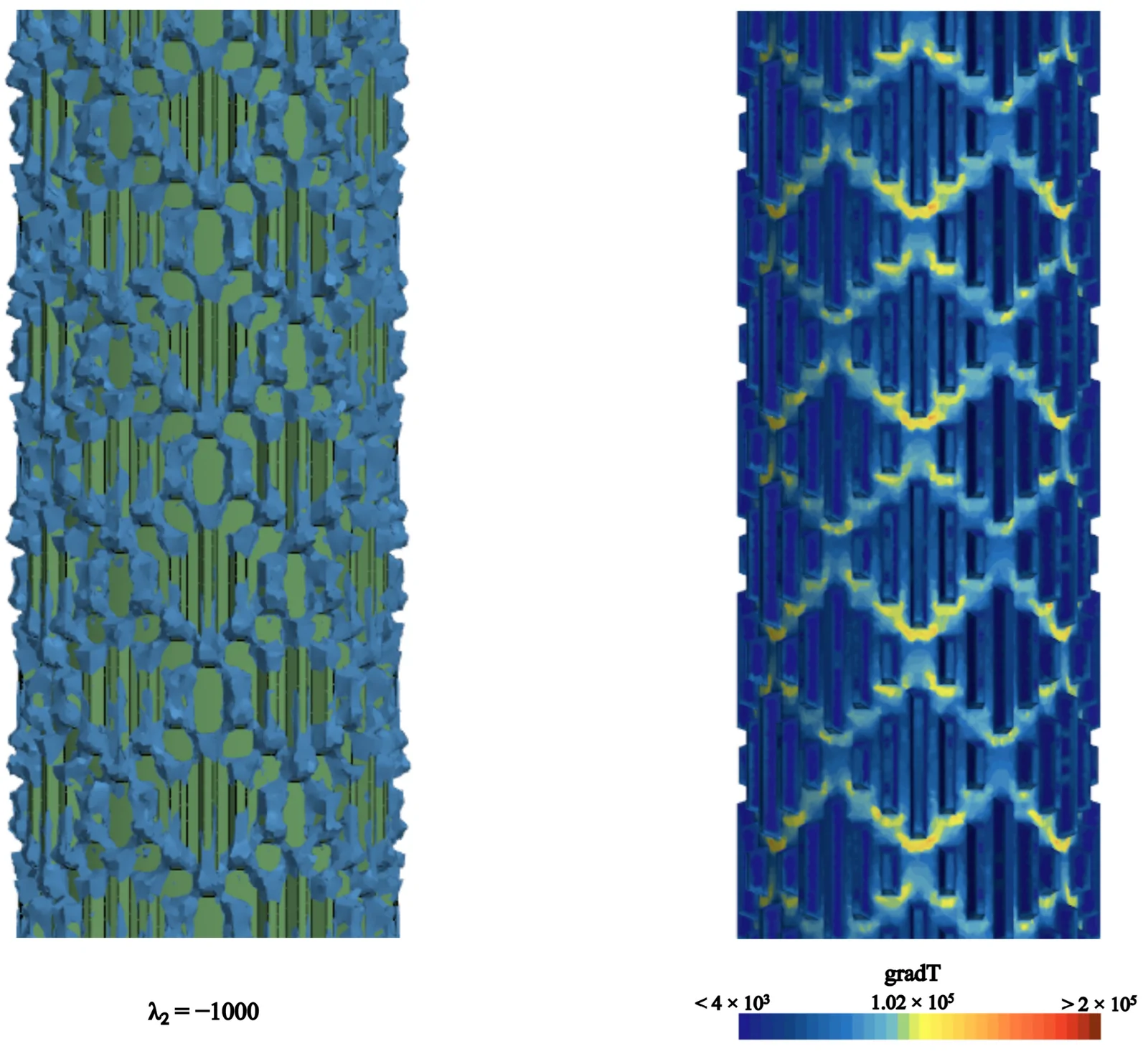
Vortex isosurfaces and temperature gradient contours of the enhanced tube.

All 32 configurations in the orthogonal array exhibit heat transfer enhancement. Relative to the smooth tube, the shark-skin-structured tube raises the heat transfer efficiency by up to 21.7% and the pressure drop by up to 245.4%—even the weakest configuration still improves efficiency by 6.2% while increasing pressure drop by 4.9%. Under identical operating conditions and working fluid, Nu is proportional to the heat transfer power Q and the friction factor f is normalized to the smooth-tube value, so the dimensionless Nu and f reproduce these same relative changes. For the typical operating condition, the area-averaged convective heat transfer coefficient on the ribbed outer surface is h_o_ = 5.67 × 10^3^ W·m^−2^·K^−1^. To examine the enhancement mechanism, configuration 7—the one giving the maximum efficiency gain—was selected for detailed analysis. Figure 4 shows the three-dimensional vortex isosurface (isovalue −1000) and temperature gradient field of this tube. By contrast, the smooth circular tube develops only sparse, flattened vortices confined to a thin near-wall layer. This indicates a comparatively stable boundary layer in which near-wall turbulent fluctuations are strongly damped, limiting the lateral mixing of hot and cold fluids.

By observing the three-dimensional vortex isosurface plot of the shark-skin-inspired heat exchange tube, a large number of dense and complex vortex structures emerge on its surface. This phenomenon indicates that the bionic microstructures significantly alter the velocity field distribution in the near-wall region, inducing strong streamwise vortices and horseshoe vortices. Such intense and complex vortex motion is a prerequisite for enhanced heat transfer. The three-dimensional vortices induced by the shark-skin-inspired structure generate strong shear effects when approaching the wall, disrupting the originally stable thermal boundary layer. The dense vortex structures promote sufficient convective mixing between the cold fluid in the main flow and the hot fluid near the wall, thereby accelerating the transport of heat from the tube wall to the main flow region. Figure 4 shows that the core regions of the secondary flow vortices spatially correspond to the peak temperature gradient zones; conversely, the temperature gradient is significantly lower at the periphery of the vortex structures. Moreover, by observing the arrangement of high-gradient contours, it can be inferred that this enhanced heat transfer does not occur uniformly but exhibits a periodic enhancement effect along with the geometric features of the surface microstructures. This spatial correspondence directly reveals the heat transfer enhancement mechanism of the vortices.

### 4.2. Effects of Geometric Parameters on Flow and Heat Transfer Performance

In the main effects plots the abscissa represents the discrete levels of each geometric factor rather than a continuous variable, so the level-averaged response is not necessarily monotonic. The difference between adjacent levels is the main effect of the factor itself, which forms the basis of the range analysis.

#### 4.2.1. Range Analysis of Average Pressure Drop

**Figure 5:**
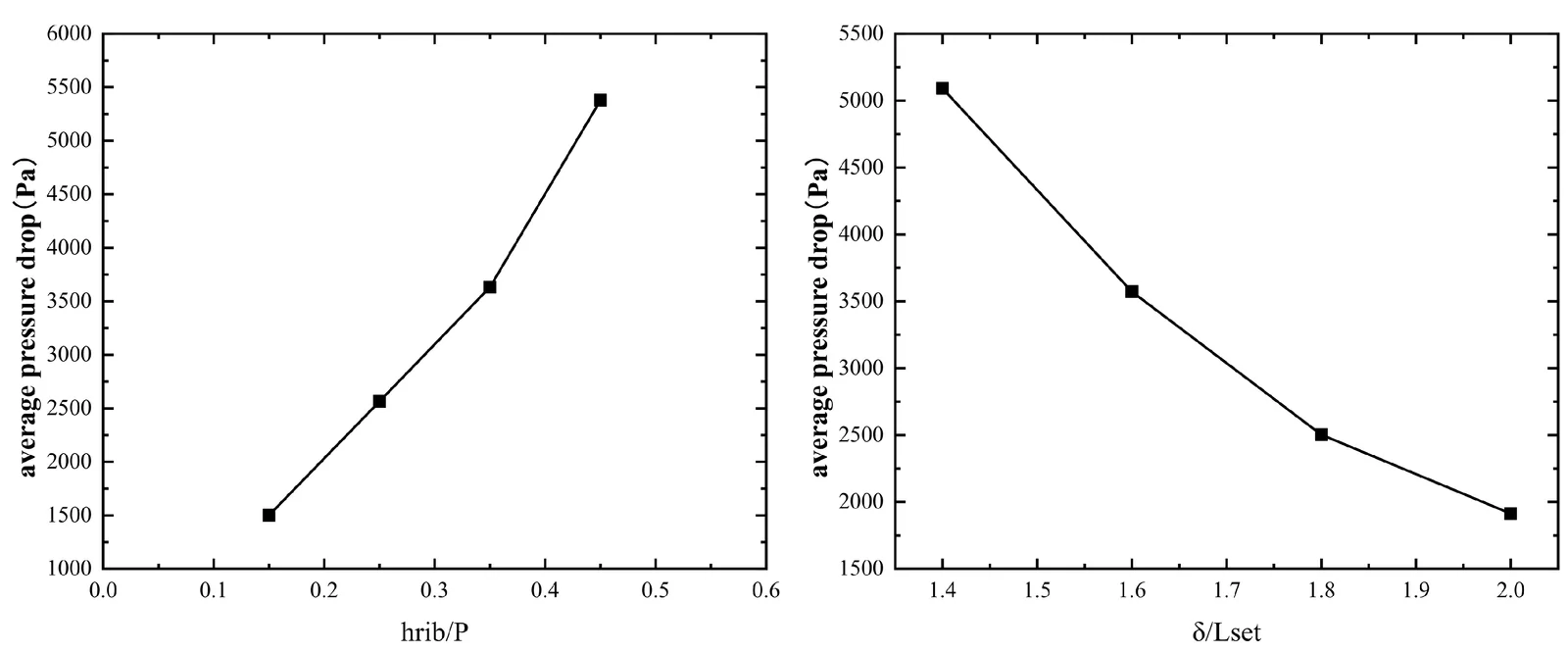

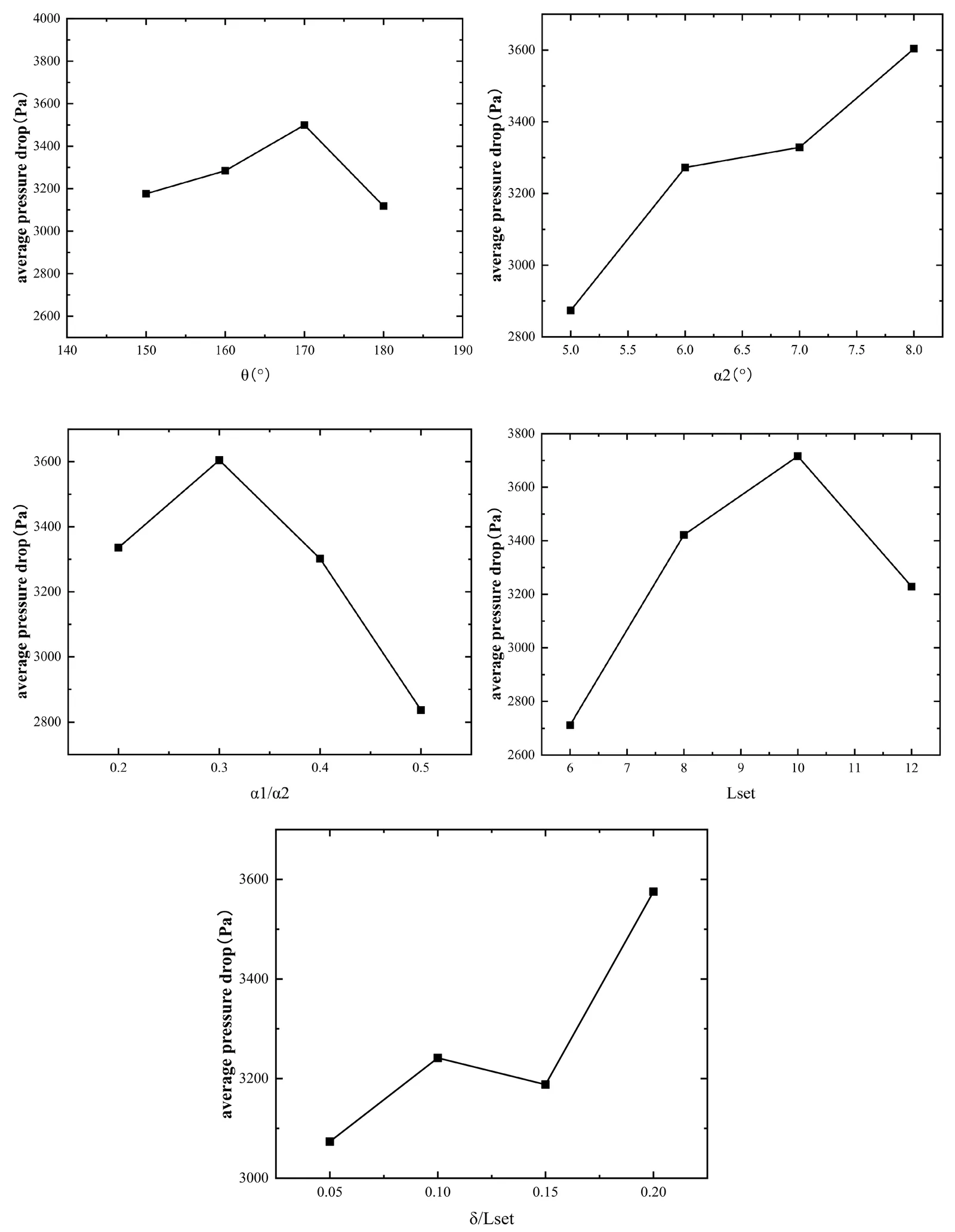
Main effects plot of average pressure drop for seven factors and four levels.

The level responses of the average pressure drop at each level of the seven factors are listed in Table 4, and the range analysis of the average pressure drop is shown in Table 5. The main effects plot of average pressure drop at each level of the seven factors is shown in Figure 5. It can be seen that the parameter hrib/P is the main influencing factor of the average pressure drop. As the value of hrib/P changes from 0.15 to 0.45, its average value shows a gradually decreasing trend with a relatively large magnitude. The parameter *θ* is the weakest influencing factor, and its average pressure drop shows a trend of first increasing and then decreasing, reaching a maximum of 3499.74 Pa at level 3.

#### 4.2.2. Range Analysis of Average Heat Transfer Power

**Figure 6:**
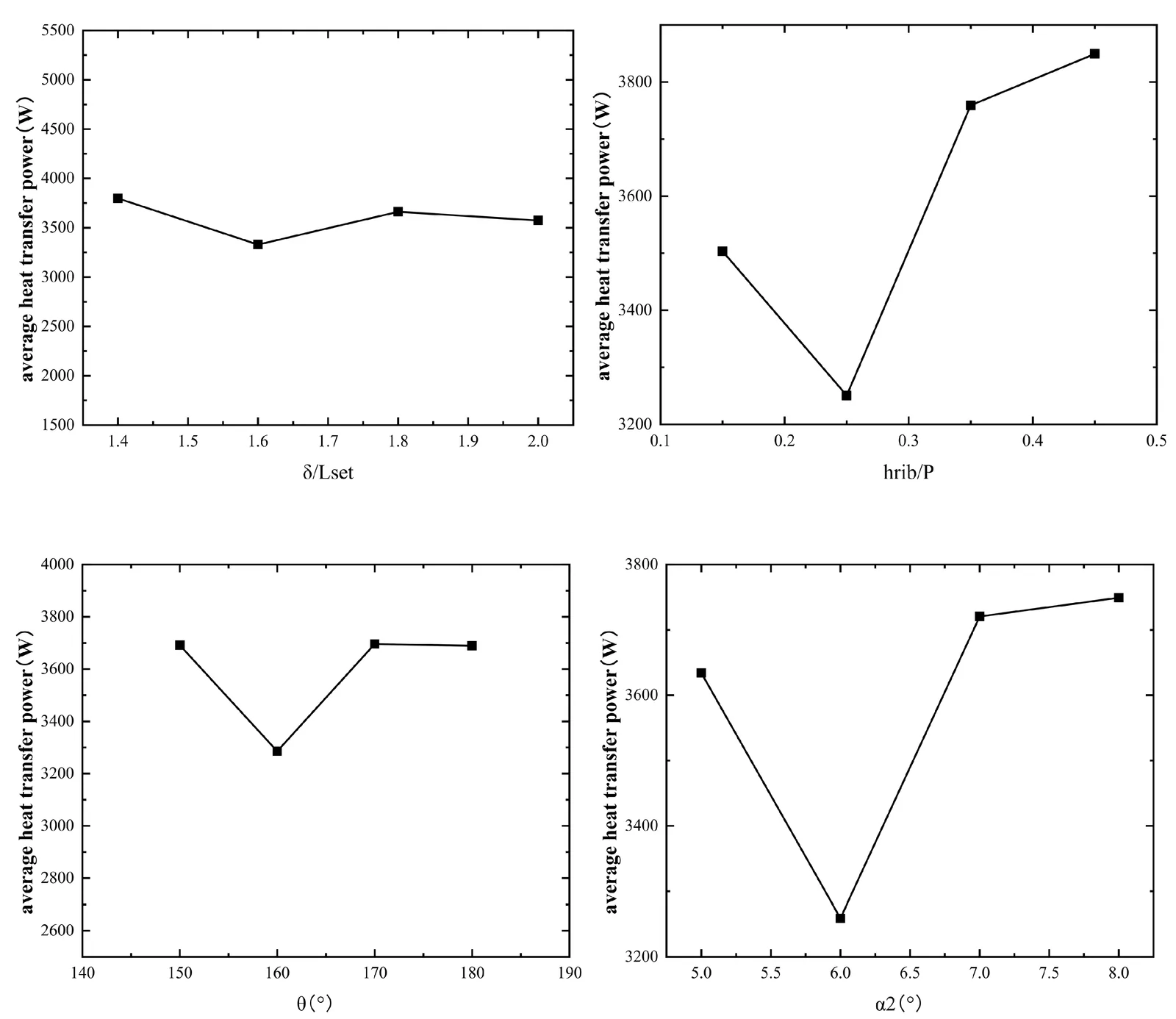

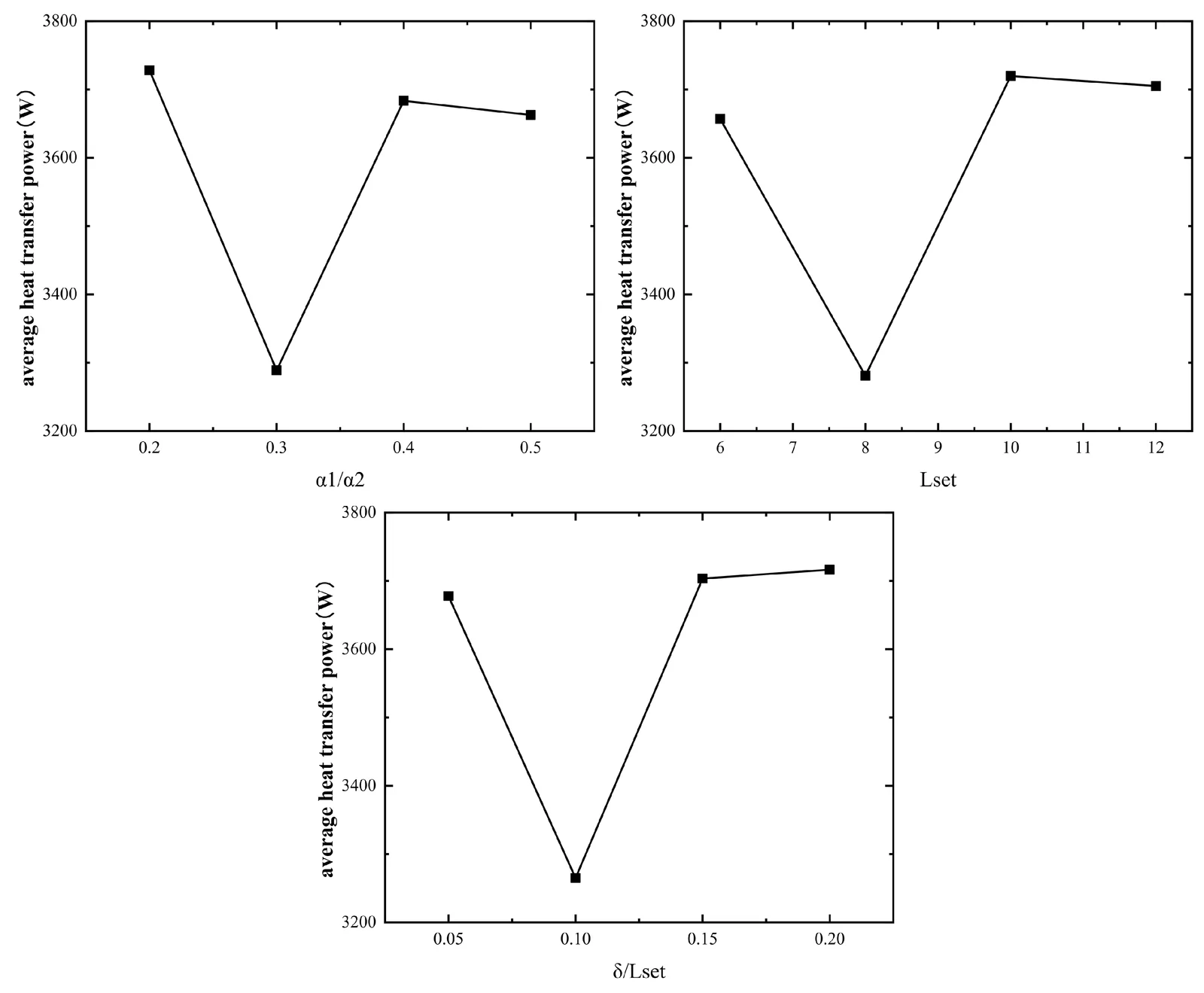
Main effects plot of average heat transfer power for seven factors and four levels.

The level responses of the average heat transfer power at each level of the seven factors are listed in Table 6. The range analysis of the average heat transfer power is shown in Table 7, and the main effects plot of the average heat transfer power at each level of the seven factors is shown in Figure 6. It can be seen that the parameter hrib/P is the main influencing factor of the average heat transfer power. As the value of hrib/P changes from 0.15 to 0.45, its average heat transfer power shows a gradually decreasing trend with a relatively large magnitude, reaching a maximum of 3798.02 W at level 1. The parameter θ is the weakest influencing factor, and its average heat transfer power shows a trend of first decreasing and then increasing, reaching a maximum of 3696.30 W at level 3. The influence of the variation in each parameter value on the average heat transfer power is generally smaller than that on the average pressure drop; especially for parameters P and hrib/P, the variation has a far greater impact on the flow pressure drop than on the heat transfer power.

### 4.3. Effect of the Shark Skin Structure on Thermal Stress Distribution

**Figure 7:**
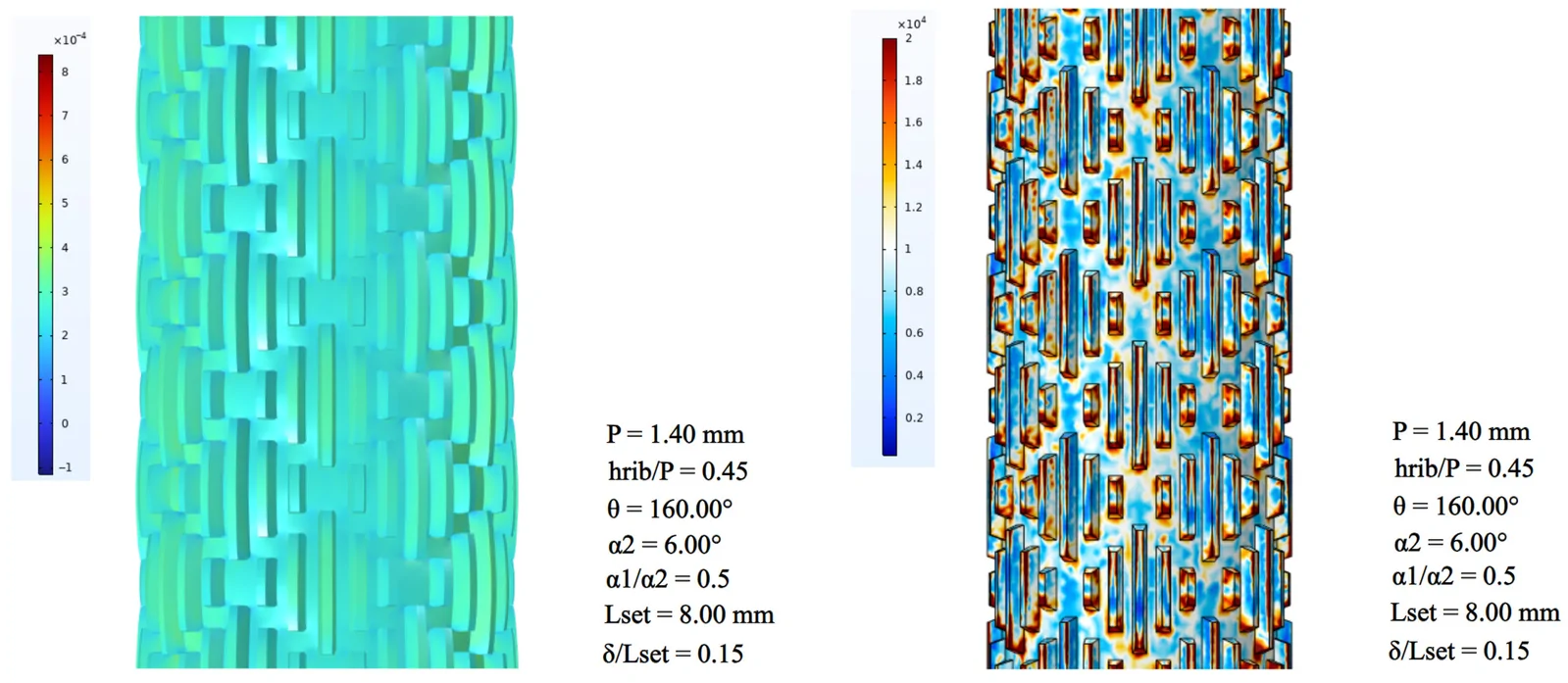
Comparison of thermal strain and temperature gradient for the shark skin heat transfer tube.

The surface structure of the shark-skin-inspired tube is complex. Although the operating temperature of the external ribs of the heat exchange tube is not high, a large temperature gradient still exists at the rib roots due to the enhanced heat transfer efficiency. Temperature gradient is a key factor affecting strain and stress. Figure 7 shows the temperature gradient and the corresponding thermal strain under specific operating conditions. The strain distribution is similar to that of the temperature gradient. High temperature gradient zones are mainly concentrated at the tips, side edges of the bionic ribs, and the transition area between the ribs and the tube base, with the highest temperature gradient occurring at the rib tips and roots. This indicates that the micro-rib structure significantly disrupts the near-wall thermal boundary layer, causing flow separation and reattachment. This flow disturbance effectively enhances the mixing of cold and hot fluids near the wall, leading to a substantial increase in the local heat transfer coefficient, whereas the low-velocity/stagnation zones on the base surface and between the ribs exhibit a lower temperature gradient.

**Figure 8:**
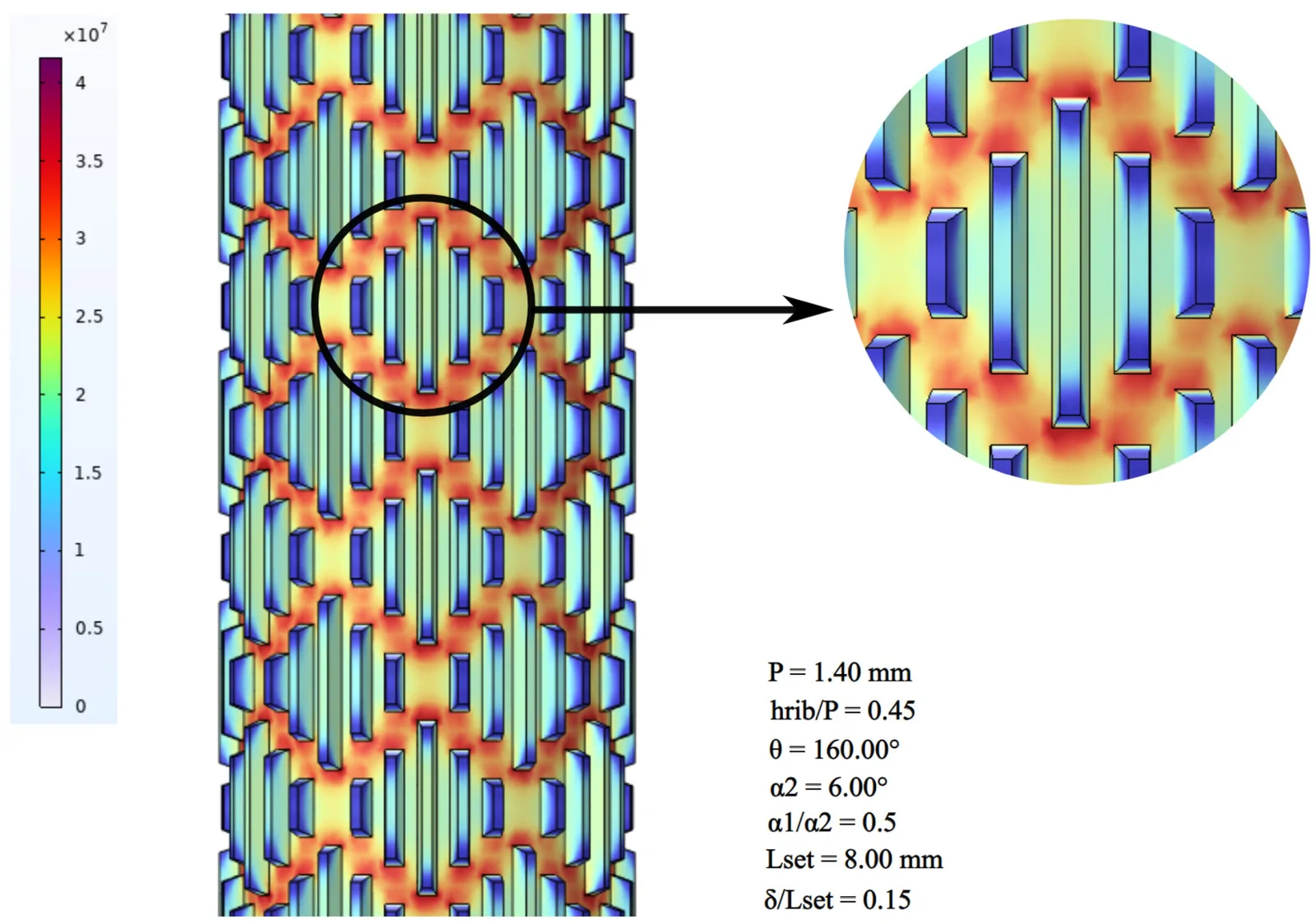
Thermal stress distribution in the shark skin heat transfer tube.

Figure 8 shows the thermal stress distribution contour of the heat exchange tube under typical operating conditions. From the thermal–structural coupling analysis results, it can be seen that the high-stress regions do not appear at the tips of the ribs but are concentrated at the interfaces between the rib roots and the base, as well as on the tube wall surface between the ribs. There are two main reasons for this distribution characteristic: first, the significant non-uniform temperature field in the radial and axial directions within the microstructure leads to incompatible thermal expansion of the material; second, the geometric discontinuity at the connection between the ribs and the base results in an obvious stress concentration effect. Under periodic start–stop or temperature fluctuation conditions, these high-stress areas are prone to become the initiation sites of fatigue cracks. Therefore, in the process of structural design and manufacturing, the design of the root fillet radius of the ribs and the thermal compatibility of the materials will be the key factors determining the service life of this heat exchange tube. Since the typical operating condition of this heat exchange tube is a low temperature difference of 40 °C, the calculated maximum equivalent stress (*σ_vm_*) is about 42 MPa, which is far below the yield limit of TA2 (the standard yield limit at room temperature is greater than 320 MPa). Therefore, under the current conditions, the heat exchange tube is entirely in the linear elastic deformation stage, without the risk of plastic yielding.

## 5. Conclusions

Based on the fluid–thermal–structural coupling method, the flow and heat transfer characteristics of the shark-skin-structured enhanced tube, the influence of geometric parameters on thermohydraulic performance, and the distribution of thermal stress on the microstructured surface were investigated. The main conclusions are as follows:

1. Compared with the smooth tube, the shark-skin-structured tube disrupts the boundary layer and induces numerous rhombic vortex structures, which improves the heat transfer efficiency by approximately 21.73%, while the flow pressure drop increases by 245.41%. This indicates a substantial enhancement in heat transfer accompanied by a considerable pressure drop penalty.
2. A range analysis was conducted on seven factors at four levels. The parameter hrib/P is the dominant factor for both the average pressure drop and the average heat transfer power. Both parameters reach their maximum values when hrib/P is at level 1. Notably, the variation of hrib/P has a far greater impact on the average pressure drop than on the heat transfer power, highlighting the need for a careful trade-off in design optimization.
3. A thermal–mechanical coupling analysis of the shark-skin-structured tube was performed, and the relationships among thermal strain, thermal stress, and temperature gradient were revealed. The stress concentration regions overlap with the zones of intensified heat transfer, demonstrating a coupling effect between thermal stress distribution and the heat transfer enhancement mechanism. This suggests that the rib roots, where high thermal gradients and stress concentration coexist, are critical regions for long-term structural integrity.

## Figures and Tables

**Table 1:** Grid independence verification.

Mesh	Total Cells	Average Nu	Relative Change	Pressure Loss Δ*P* (Pa)	Relative Change
Mesh 1	2.14 million	172.5	—	245.8	—
Mesh 2	3.42 million	181.3	5.10%	258.4	5.13%
Mesh 3	4.86 million	184.6	1.82%	262.1	1.43%
Mesh 4	6.15 million	185.7	0.62%	263.3	0.45%

**Table 2:** Design parameters of the shark skin heat transfer tube.

Limits	P	h_rib_/P	θ	α_2_	α_1_/α_2_	L_set_	δ/L_set_
Lower bound	1.5 mm	0.15	150°	5°	0.2	6 mm	0.05
Upper bound	2.0 mm	0.45	180°	8°	0.5	12 mm	0.2

**Table 3:** 32 orthogonal array geometric parameters of shark-skin-microstructured heat exchange tubes.

Case	*P*	*h_rib_*/*P*	*θ*	*α_2_*	*α_1_*/*α_2_*	*L_set_*	*δ*/*L_set_*
1	1.40 mm	0.15	180.00°	5.00°	0.2	6.00 mm	0.05
2	1.40 mm	0.15	170.00°	6.00°	0.4	12.00 mm	0.20
3	1.40 mm	0.25	160.00°	8.00°	0.4	6.00 mm	0.10
4	1.40 mm	0.25	150.00°	7.00°	0.2	12.00 mm	0.15
5	1.40 mm	0.35	180.00°	7.00°	0.3	8.00 mm	0.20
6	1.40 mm	0.35	170.00°	8.00°	0.5	10.00 mm	0.05
7	1.40 mm	0.45	160.00°	6.00°	0.5	8.00 mm	0.15
8	1.40 mm	0.45	150.00°	5.00°	0.3	10.00 mm	0.10
9	1.60 mm	0.15	160.00°	8.00°	0.3	10.00 mm	0.20
10	1.60 mm	0.15	150.00°	7.00°	0.5	8.00 mm	0.05
11	1.60 mm	0.25	180.00°	5.00°	0.5	10.00 mm	0.15
12	1.60 mm	0.25	170.00°	6.00°	0.3	8.00 mm	0.10
13	1.60 mm	0.35	160.00°	6.00°	0.3	12.00 mm	0.05
14	1.60 mm	0.35	150.00°	5.00°	0.2	6.00 mm	0.20
15	1.60 mm	0.45	180.00°	7.00°	0.4	12.00 mm	0.10
16	1.60 mm	0.45	170.00°	8.00°	0.2	6.00 mm	0.15
17	1.80 mm	0.15	160.00°	5.00°	0.5	12.00 mm	0.10
18	1.80 mm	0.15	150.00°	6.00°	0.3	6.00 mm	0.15
19	1.80 mm	0.25	180.00°	8.00°	0.3	12.00 mm	0.05
20	1.80 mm	0.25	170.00°	7.00°	0.5	6.00 mm	0.20
21	1.80 mm	0.35	160.00°	7.00°	0.4	10.00 mm	0.15
22	1.80 mm	0.35	150.00°	8.00°	0.2	8.00 mm	0.10
23	1.80 mm	0.45	180.00°	6.00°	0.2	10.00 mm	0.20
24	1.80 mm	0.45	170.00°	5.00°	0.4	8.00 mm	0.05
25	2.00 mm	0.15	170.00°	8.00°	0.4	8.00 mm	0.15
26	2.00 mm	0.15	160.00°	7.00°	0.2	10.00 mm	0.10
27	2.00 mm	0.25	170.00°	5.00°	0.2	8.00 mm	0.20
28	2.00 mm	0.25	160.00°	6.00°	0.4	10.00 mm	0.05
29	2.00 mm	0.35	180.00°	6.00°	0.5	6.00 mm	0.10
30	2.00 mm	0.35	170.00°	5.00°	0.3	12.00 mm	0.15
31	2.00 mm	0.45	160.00°	8.00°	0.3	6.00 mm	0.05
32	2.00 mm	0.45	150.00°	7.00°	0.5	12.00 mm	0.20

**Table 4:** Level response of shark skin microstructure heat transfer tube geometric parameter (ΔP).

Type	Level	p	h_rib_/P	θ	α_2_	α_1_/α_2_	L_set_	δ/L_set_
Κ_ΔP_	1	40,730.13	12,007.84	25,408.76	22,989.11	26,686.20	21,696.37	24,588.32
Κ_ΔP_	2	28,575.22	20,517.69	26,276.60	26,180.17	28,838.00	27,375.11	25,932.39
Κ_ΔP_	3	20,020.11	29,066.69	27,997.89	26,629.00	26,413.33	26,643.64	25,504.56
Κ_ΔP_	4	15,303.25	43,036.48	24,945.45	28,830.43	22,691.18	25,830.58	28,603.43
k_ΔP_	1	5091.27	1500.98	3176.10	2873.64	3335.77	2712.05	3073.54
k_ΔP_	2	3571.90	2564.71	3284.57	3272.52	3604.75	3421.89	3241.55
k_ΔP_	3	2502.51	3633.34	3499.74	3328.62	3301.67	3715.83	3188.07
k_ΔP_	4	1912.91	5379.56	3118.18	3603.80	2836.40	3228.82	3575.43

**Table 5:** Range of shark skin microstructure heat transfer tube geometric parameters on pressure drop.

R	p	h_rib_/P	θ	α_2_	α_1_/α_2_	L_set_	δ/L_set_
R_ΔP_	3178.36	3878.58	381.55	730.17	768.35	1003.78	501.89

**Table 6:** Level response of shark skin microstructure heat transfer tube geometric parameter (Q).

Type	Level	P	h_rib_/P	θ	α_2_	α_1_/α_2_	L_set_	δ/L_set_
Κ_Q_	1	30,384.17	28,028.01	29,532.90	29,073.51	29,823.66	29,255.35	29,422.73
Κ_Q_	2	26,633.45	26,005.12	26,284.02	26,070.78	26,309.80	26,247.27	26,120.71
Κ_Q_	3	29,297.50	30,073.52	29,570.43	29,764.03	29,467.44	29,757.63	29,626.68
Κ_Q_	4	28,586.49	30,794.96	29,514.25	29,993.29	29,300.70	29,641.36	29,731.48
k_Q_	1	3798.02	3503.50	3691.61	3634.19	3727.96	3656.92	3677.84
k_Q_	2	3329.18	3250.64	3285.50	3258.85	3288.73	3280.91	3265.09
k_Q_	3	3662.19	3759.19	3696.30	3720.50	3683.43	3719.70	3703.34
k_Q_	4	3573.31	3849.37	3689.28	3749.16	3662.59	3705.17	3716.43

**Table 7:** Range of shark skin microstructure heat transfer tube geometric parameters on heat transfer power.

R	p	h_rib_/P	θ	α_2_	α_1_/α_2_	L_set_	δ/L_set_
R_Q_	468.84	598.73	410.80	490.31	439.23	438.79	451.35

## Data Availability

The original contributions presented in this study are included in the article. Further inquiries can be directed to the corresponding author.

## Nomenclature

D	diameter of the tube, mm
L	test section length, m
Nu	Nusselt number, -
∆P	pressure drop, Pa
Re	Reynolds number, -
T	temperature, K
B	field synergy angle, ◦
Hrib	height of ribs, mm
P	streamwise rib spacing, mm
Lset	length of a set of ribs, mm
α1	apex angle of ribs, ◦
α2	base angle of ribs, ◦
θ	cross-sectional included angle, ◦
δ	spacing between adjacent ribs, mm
σ_vM_	von Mises equivalent stress, Mpa
SST	k-ω shear stress transport k-ω turbulence model, -
